# Insights into innate immune cell evasion by *Chlamydia trachomatis*

**DOI:** 10.3389/fimmu.2024.1289644

**Published:** 2024-01-25

**Authors:** Xinglv Wang, Hongrong Wu, Chunxia Fang, Zhongyu Li

**Affiliations:** Institute of Pathogenic Biology, Hunan Provincial Key Laboratory for Special Pathogens Prevention and Control, School of Nursing, Hengyang Medical College, University of South China, Hengyang, China

**Keywords:** *Chlamydia trachomatis*, innate immune cells, innate immunity, immune evasion, survival and growth

## Abstract

*Chlamydia trachomatis*, is a kind of obligate intracellular pathogen. The removal of *C. trachomatis* relies primarily on specific cellular immunity. It is currently considered that CD4^+^ Th1 cytokine responses are the major protective immunity against *C. trachomatis* infection and reinfection rather than CD8^+^ T cells. The non-specific immunity (innate immunity) also plays an important role in the infection process. To survive inside the cells, the first process that *C. trachomatis* faces is the innate immune response. As the “sentry” of the body, mast cells attempt to engulf and remove *C. trachomatis*. Dendritic cells present antigen of *C. trachomatis* to the “commanders” (T cells) through MHC-I and MHC-II. IFN-γ produced by activated T cells and natural killer cells (NK) further activates macrophages. They form the body’s “combat troops” and produce immunity against *C. trachomatis* in the tissues and blood. In addition, the role of eosinophils, basophils, innate lymphoid cells (ILCs), natural killer T (NKT) cells, γδT cells and B-1 cells should not be underestimated in the infection of *C. trachomatis*. The protective role of innate immunity is insufficient, and sexually transmitted diseases (STDs) caused by *C. trachomatis* infections tend to be insidious and recalcitrant. As a consequence, *C. trachomatis* has developed a unique evasion mechanism that triggers inflammatory immunopathology and acts as a bridge to protective to pathological adaptive immunity. This review focuses on the recent advances in how *C. trachomatis* evades various innate immune cells, which contributes to vaccine development and our understanding of the pathophysiologic consequences of *C. trachomatis* infection.

## 1 Introduction

*C. trachomatis*, a specific intracellular pathogen, is closely associated with human epidemic diseases. According to the World Health Organization, in 2020, an estimated 129 million people would be infected with *C. trachomatis* (1). There are 19 serovars of *C. trachomatis*. In addition to trachoma, inclusion conjunctivitis, and infantile pneumonia(serovars A to C), it also causes more serious genitourinary tract infections(serovars D to K) and lymphogranuloma venereum(serovars L). The former is highly curable and can be treated with large-scale azithromycin (2), while the latter is insidious and chronic both in men and women (3). Although sensitive to antibiotics, their therapeutic benefit is limited mainly because of silent (asymptomatic) and recurrent infections attributable to immune evasion and the development of partial immunity from these infections. In the long term, these recurrent infections are thought to be contributing to the development of pelvic inflammatory disease (4), tubal infertility (5), cervical cancer (6), adverse pregnancy, miscarriage (7) in women, urethritis, epididymitis, orchitis, prostatitis, proctitis (8) or reactive arthritis (9) in men. These outcomes are thought to be culminating either alone or as a complex combination of Chlamydial pathogenesis, deficient or inferior immunological memory- immune escape and immunopathology. *Chlamydia* lives in membrane-coated vacuoles, called inclusion, which protect it from the humoral immune response. It has been found that *Chlamydia* obtains nutrients (amino acids, lipids (10), iron (11)) mainly from the host cell, and acquires ATP (12) entirely from the host. *Chlamydia* takes self-serving measures to deal with nutritional crisis, one of which is to limit the detection of innate immunity (13). NF-κB plays an important role in the inflammatory response. Surprisingly, no obvious signs of NF-κB activation were detected in *Chlamydia*-infected cells (14). *C. trachomatis* blocks NF-κB signaling through effector ChlaDUB1 reversal of IκB-α ubiquitination (15) and CPAF-mediated p65/RelA degradation (16). However, the *in vivo* role of CPAF in NF-κB signaling has not been proven. Inflammatory damage caused by chlamydial infection is largely due to both innate and adaptive immunity. Chlamydial infection stimulates host cells to produce interleukins, interferons, and tumor necrosis factor, which play a dual role in infection (17, 18). Consequent to infection of the upper genital tract, infiltration of neutrophils and monocytes that are responsible for potentially deleterious inflammation along with the bystander T cells both with the potential to cause immunopathology (19). Highly potent antibodies may also cause corresponding immunopathology through mechanisms such as activation of complement (20), ADCC (antibody-dependent cellular cytotoxicity) (21), and the emergence of a type IV hypersensitivity reaction (22). In addition, immune escape of *Chlamydia* is also an important reason for chronic recurrent infections, which will be described primarily in this review.

Recent studies indicate that *C. trachomatis* uses effector molecules like GarD that helps it to escape immune surveillance via avoiding ubiquitination and proteosomal degradation (23, 24). Recent findings indicate that Chlamydial lipopolysaccharide (LPS) (25) and lipooligosaccharide (LOS) (26) do not readily trigger innate inflammatory pathways, thus avoiding early innate recognition and promoting its survival and multiplication.

In summary, the key to maintaining the intracellular survival and persistence of *C. trachomatis* is to escape from the host’s innate immune cells. In recent years, it has been found that properly functioning immune cells have the potential to treat disease, and immune responses are often associated with the course of disease. When the body is in the midst of a persistent infection or the immune cells are not functioning properly, the ability of the immune system to clear the infection is declining. Then a critical point is reached where the immune cells are unable to clear the infected cells, and the disease occurs. The development of a novel vaccine against *C. trachomatis* infection benefits greatly from research on cancer immunotherapy (27) and experimental vaccinations against intracellular diseases. In fact, after H Su et al. immunized mice intravenously with bone marrow-derived dendritic cells stimulated *in vitro* by dead *chlamydia*, DC was able to efficiently perform its functions of bacterial phagocytosis and antigen presentation. The results showed that this method of immunization produced protective immunity against *Chlamydia* infection in the female genital tract comparable to that following *in vivo* infection (28). Karunakaran et al. have employed DCs pulsed with chlamydial immunopeptides in immunizations by adoptive transfer. Immunized mice developed Th1 protective immunity and partially resisted chlamydial lung and genital infections (29). However, there is a long way to go in the development of immune cell-targeted biologics in chlamydial immunity and immunopathology for prevention or treatment (30, 31).

## 2 Pathogenesis and immunity

Unlike other bacteria, *C. trachomatis* has a unique biphasic developmental cycle. In the initial step, non-replicating elementary bodies (EBs) bind to the host acetyl heparan sulfate proteoglycan (HSPG) and primarily the receptor tyrosine kinase (RTK), injecting various effector proteins called *C. trachomatis* secretory proteins (CtSPs) into the cytoplasm via the type III secretion system (T3SS) or other secretory mechanisms (32, 33). After entry, they differentiate into non-infectious replicating reticulate bodies (RBs) within parietal vesicles called inclusions, and RB-secreted inclusions membrane proteins (Incs) are incorporated into the membranes of the compartment (34). The interaction of *C. trachomatis* proteins with host proteins involves altering vesicular transport in extracellular vesicles, regulating cell survival pathways, and suppressing the innate host immune response. The Chlamydial effector proteins interfere with the host’s innate immune response, such as INCs, TepP (35), CPAF, Pgp3 (36), Pgp4 (37) and 60 other proteins (38), which promote intracellular survival of *Chlamydia* and limit the host response to infection. RBs continue to differentiate in inclusions and, at later stages, asynchronously undergo secondary differentiation to create new EBs. Studies have shown that *C. trachomatis* divides by a polarized budding mechanism, rather than binary fission (39). In the final process, intracellular EBs are release by two pathways described so far: lysis cell destruction or exit by extrusion formation. The particular form of cyclic propagation from EB to RB to EB occurs repeatedly in neighboring cells of the host, which takes approximately 36-48h to complete a developmental cycle.

The first line of defense in the human immune system is the skin and mucous membranes, and the second line is the phagocytes and bactericidal substances in the body fluids, which together constitute innate immunity. *C. trachomatis* genital serovars have a tropism for columnar epithelial cells located on mucosal surfaces (40, 41). On the one hand, host epithelial cells recognize the invasion of *C. trachomatis* antigens by surface receptors, endosomal receptors, and innate immune factors. These antigens are first blocked by the mucosal barrier and neutralized by mucosal antibodies. Microbiota namely lactobacillus along with lactoferrin confer an important mucosal defense in the cervicovaginal region (42). Upon breach of this barrier, the infected cells release cytokines and chemokines (43) that serve to recruit cells like neutrophils and monocytes as well as others that serve to curtail the infection and limit its spread (44). Macrophages engulf the bacteria and produce pro-inflammatory factors (45); IFN-γ secreted by NK cells not only kills infected host cells but also induces an immune response to Th1 (46, 47); When infection leads to the development of antigen-specific immunity, CD4^+^ T cells along with B cells produce immunity where *chlamydia*-specific Th1 CD4^+^ T cells and antibodies are considered protective (48, 49), whereas CD8^+^ T cell response is considered non-essential or even pathogenic (50, 51). These cells interact and collaborate to clear *C. trachomatis*.

On the other hand, this infection stimulates the establishment of immunogenicity is necessary to generate a good protective immune response. *Chlamydia* has evolved to evade immunity as well as actively subvert it by inhibiting the cytokines and chemotactic proteins produced by epithelial cells (52, 53). It interferes with the antigen-presenting function of antigen-presenting cells (APCs) (downregulation of MHC class I and II molecules) (54, 55), regulating specific cytokines with multiple effects (IL-18, IFN-γ, TNF-α), and anti-apoptosis (increased cell survival signaling and CPAF release) (56–58). In addition, recent studies have shown that intracellular RBs can enter uninfected neighboring cells via tunneling nanotubes (TNT) (59). This allows *Chlamydia* to remain unexposed to body fluids, thus evading to some extent the pursuit of various cytokines in body fluids.

## 3 Evasion of classical innate immune cells

### 3.1 Mφ

**Figure 1:**
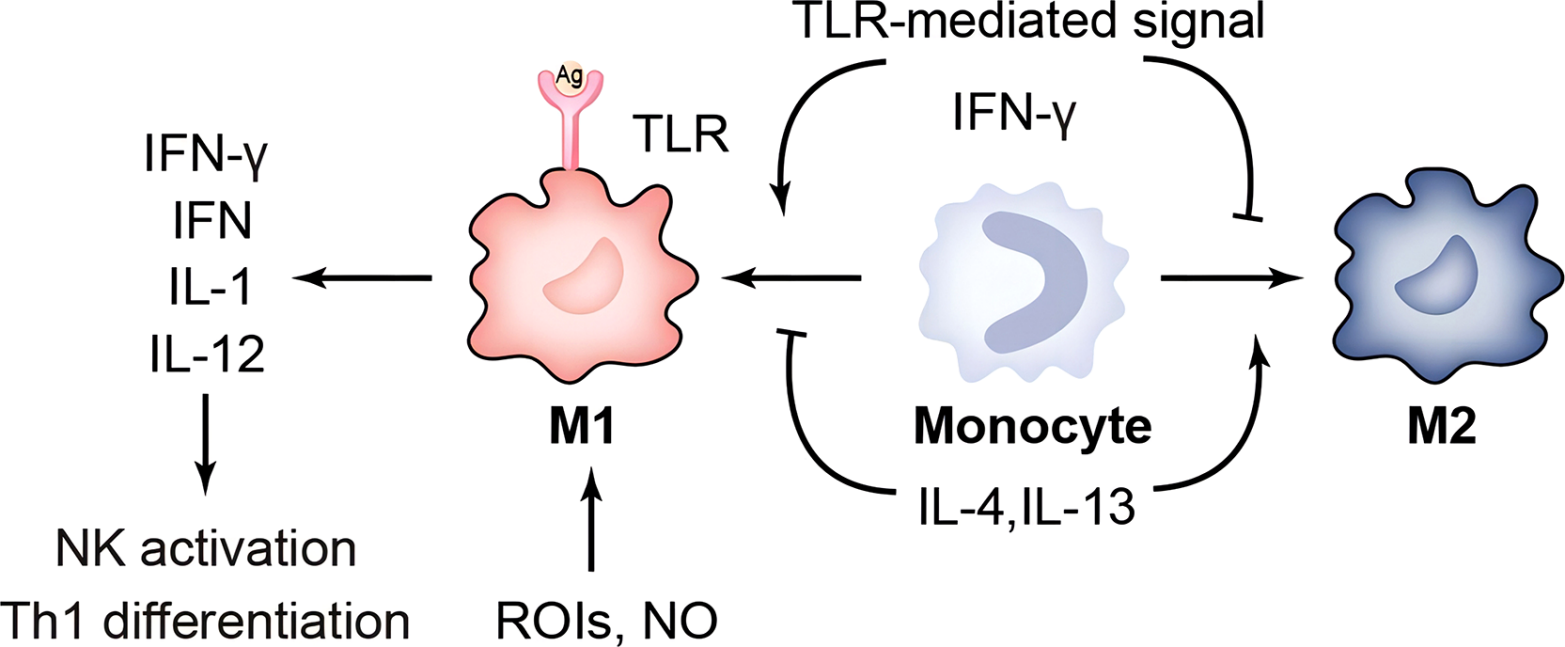
Immune response of macrophages. TLR, toll-like receptor; ROIs, reactive oxygen intermediates; IFN-γ, Interferon gamma; IL-1, interleukin-1.

Tissue macrophages (Mφ) generally engulf bacteria early in the infection when they are outside the host cells. It is known that *C. trachomatis* entry into the monocyte macrophages is less efficient when compared to epithelial cells. Moreover, ROS-mediated microbicidal activity is unable to kill the engulfed bacteria, and only IFN-γ stimulated, highly activated phagocytes with sufficient effector molecules can effectively kill bacteria (17, 60) (**Figure 1**). Experiments by Chen, et al. in 2017 showed that both LPS/ATP and murine chlamydial infection of mouse bone marrow-derived macrophages (BMDM) could lead to caspase-1 cleavage and IL-1β released. However, unlike LPS/ATP, the inflammatory molecular switch RIP3 was not involved in the activation of NLRP3 inflammatory vesicles in murine *Chlamydia*-stimulated BMDM, suggesting that chlamydial infection leads to caspase-1 cleavage and IL-1β release by a different mechanism than LPS/ATP (61). Two years later, further experiments showed that lipopolysaccharide (LPS) of *C. trachomatis* in BMDM, unlike other Gram-negative bacteria, blocked downstream signaling of TRIF and MyD88 classical inflammatory pathways and failed to activate non-classical inflammatory pathways mediated through caspase-11 to evade innate immunity. This study explored the mechanism in more depth: trachoma LPS, while effectively binding CD14, cannot effectively induce TLR4/MD-2 complex dimerization or endocytosis, implying that the classical pathway is blocked at its source. It is speculated that this phenomenon may be related to the fact that lipid A of chlamydial LPS is penta-acylated, rather than hexa-acylated (25).

Macrophages are divided into two phenotypes, M1 and M2: IFN-γ/LPS-induced M1 and selectively activated, IL-4-induced M2. M1 has a strong capacity to engulf and kill bacteria that triggers an inflammatory response by releasing chemokines and pro-inflammatory cytokines. And M2 cells inhibit inflammatory responses or participate in tissue repair and fibrosis. Buchacher T et al. showed that positive *Chlamydia pneumoniae* (Cpn) lipopolysaccharide staining and quantification of 16 S rDNA were significantly higher in M2 than in M1. A large number of intact perinuclear inclusions were found in M2-type macrophages, whereas rupture of inclusion bodies occurred in M1-type macrophages (62). The *Chlamydia muridarum* and murine bone-marrow derived macrophages showed that although M1 could mediate IFN-γ to control infection, it could not eliminate intracellular *chlamydia* (63). This suggests that M2-type macrophages provide a better environment for Cpn to survive. It has been shown that Nutlin-3 inhibits *Chlamydia abortus* growth and affects TNF-α secretion in M1 in a dose-dependent manner (64). In addition to improving the survival environment within macrophages, another important escape mechanism is the regulation of apoptosis. Utilizing CRISPR/Cas9 technology, Amy T.Y. Yeung and his colleagues hypothesized that deletion of the IL-10/IL-10R signaling pathway may resist macrophage apoptosis and thus sustain chlamydial growth (65). In addition, *Chlamydia* is expelled from the host cell by extrusion release, forming an inclusion-like structure called extrusions. Extrusions retard macrophage killing and eventually release infectious EB from macrophages, which facilitates the spread of EB to more distant sites. Extrusions are also able to survive longer in the extracellular environment than free *C. trachomatis* EB (66). This chlamydia-specific cell exit is likely a self-interested ability shaped by thousands of years of host-pathogen interactions. In summary, chlamydial survival in macrophages is suitable but limited. The restricted growth pattern is often associated with lysosomal trafficking, perforin-2 release, and nutrient starvation. Further work is needed to investigate the mechanisms of *chlamydia*-macrophage interactions, as well as to confirm them *in vivo*. This may, in some ways, increase the clearance susceptibility of the macrophage and decrease its ability to assist in chlamydial transmission.

### 3.2 NE

Neutrophil (NE) is the most abundant innate immune cells and the first leukocytes recruited to infected tissues. Neutrophils kill microorganisms primarily by phagocytosis and activation of the NADPH oxidase machinery (67). When neutrophils encounter pathogens, they activate their bactericidal reservoir to produce reactive oxygen species (ROS) and neutrophil extracellular traps (NETs). NETs are DNA structures decorated with cytoplasm, granules and nucleoproteins, and they can capture microorganisms including bacteria, viruses and 50 species of fungi. A variety of microorganisms are known to activate neutrophils and induce the formation of NETs. However, in order to evade or survive in neutrophils, some pathogens (68), such as *C. trachomatis*, have evolved mechanisms to degrade extracellular chromatin traps by secreting effector nucleases and proteases. It has been shown that chlamydial protease-like activating factor (CPAF) directly affects PMN survival and that formyl peptide receptor 2 (FPR2) is a key target of CPAF. In addition, *C. trachomatis* is able to reverse the short-lived neutrophils and delay apoptosis by activating ERK1/2 and PI3K/Akt survival signaling pathways (69); a key protein in human intracellular defense against *Chlamydia* is the tryptophan-degrading enzyme indoleamine 2,3-dioxygenase (IDO), but its activation does not inhibit chlamydial survival in neutrophils (70).

In pelvic inflammatory disease caused by *C. trachomatis*, interleukin1α(IL-1α) and type I interferon released upon the death of infected inflammatory cells favored the pathogen over the host. Persistent infection and chronic antigenic stimulation impair the ability of T cells to produce IFN-γ, thereby attenuating the protective response of Th1 and Th2 (4). In addition, *Chlamydia*-infected neutrophils exhibit high levels of extracellular ATP, which can act as a damage-associated molecular pattern (DAMP) to activate submucosal macrophage NLRP3 inflammatory vesicles, thereby driving damaging immunopathology (71). To some extent, these immune evasion mechanisms suggest that *C. trachomatis* infection can be overcome by the use of CPAF inhibitors, vaccines with Th1-inducing adjuvants providing antigens, *etc.*

### 3.3 DC

Classical dendritic cell (cDC) is the bridge between innate and adaptive immunity, and it is currently recognized as the most powerful specialized antigen-presenting cell in the body. From immature DC (iDC) to mature DC, it becomes progressively less capable of antigen uptake and processing (phagocytosis) and progressively more capable of antigen presentation. DCs are the sole initiators of initial T-cell activation (72). *C. trachomatis*, as an exogenous antigen, is internalized and processed by the APC, then processed by the proteasome in the cytoplasm, and finally activates CD4^+^T lymphocytes mainly through MHC-II molecules on the surface of DC. DC cells with the efficient presentation of cross-antigens activate CD8^+^T lymphocytes via surface-expressed MHC class I molecules in response to innate antigens in cells already infected with *C. trachomatis.* Moreover, CD8α^+^ dendritic cells were found to present antigen to CD8^+^T cells and induce Th1 production in mice at a better level than CD8α^-^ using the adoptive transfer method (73). It has been shown that *C. trachomatis* hijacks the DC endocytic recycling system by recruiting Rab proteins associated with the recycling pathway around the inclusions, which leads to adverse changes in MHC-I intracellular transport (54).

As previously described, *Chlamydia* exits infected host cells via extrusion formation, which confers an infectious advantage to *Chlamydia* and promotes its survival within macrophages (74). It was found that the extrusion formation also prolonged bacterial survival within dendritic cells and altered the initial innate immune signaling of dendritic cells. Protective immunity against *Chlamydia* is primarily driven by IFN-γ producing T cells. Unlike macrophages, phagocytosis of extrudates leads to rapid apoptosis of DCs through a caspase 3/7-dependent mechanism, whereas exposure to free *Chlamydia* does not undergo apoptosis, directly preventing the initiation of adaptive immune responses (75). In 2017, Khamia Ryans et al. demonstrated that alpha enolase (ENO1) deficiency affects DC survival, maturation, and antigen presentation characteristics *in vivo* and *in vitro* experiments. Thus, modulation of ENO1 facilitates the enhancement of DC function, which could be used as an immunotherapeutic strategy to generate long-term immunity against *Chlamydia*-induced tubal lesions (76). In fact, using a combination of affinity chromatography and tandem mass spectrometry, it has previously been shown that DCs transferred with the chlamydial antigenic peptide Ags: PmpG1_25-500_, RplF, or PmpE/F-2_25-575_ overtly induced significant protective immunity against pulmonary and genital tract infections. Among them, PmpG1_25-500_ is the most immunoprotective and could be a candidate for T cell protein-based subunit vaccines (77).

### 3.4 Other granulocytes

**Figure 2:**
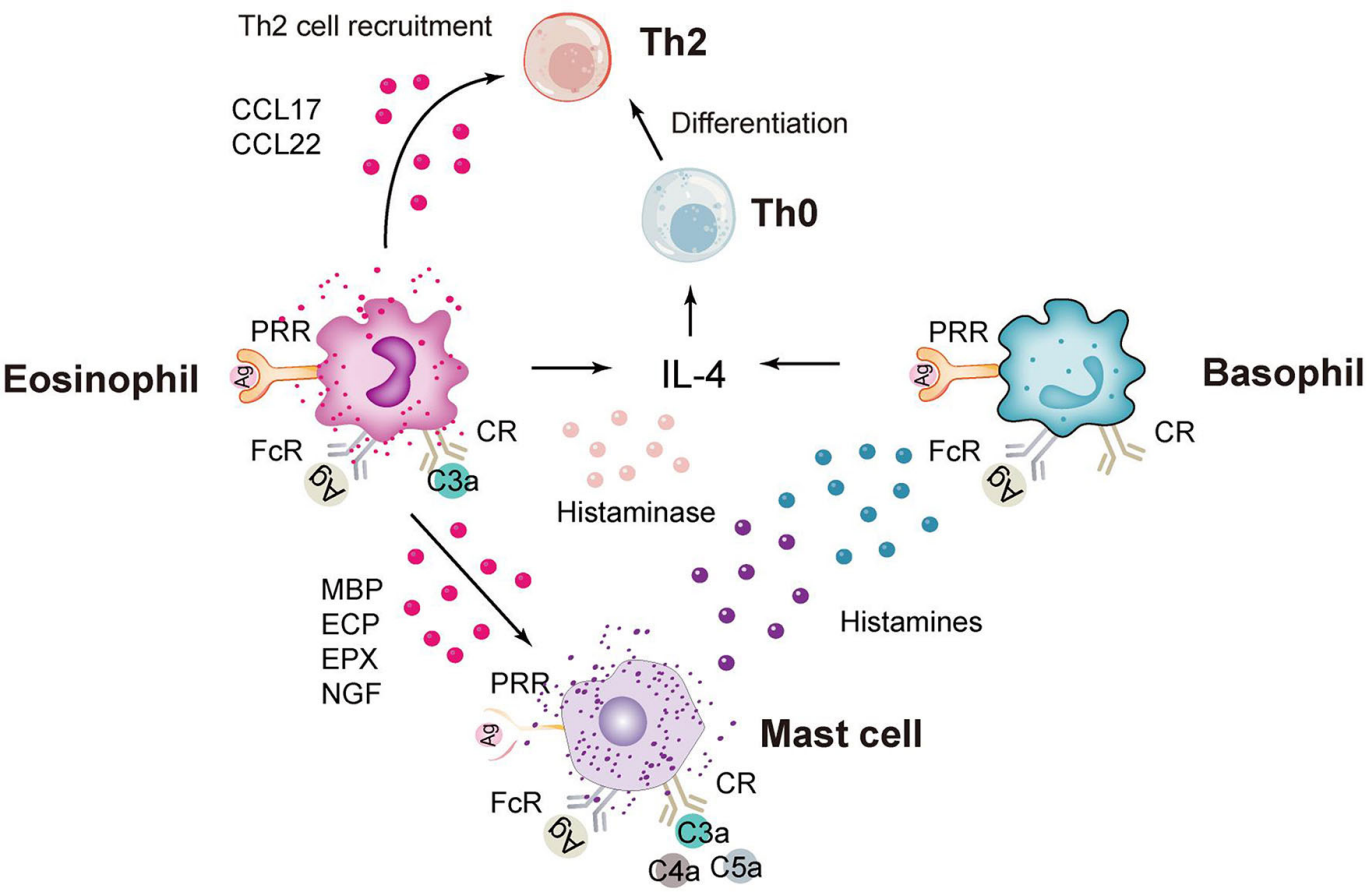
Immune response of eosinophils, basophils and mast cells. CCL17, chemokine (C-C motif) ligand 17; PRR, pattern recognition receptor; FcR, Fc receptor; CR, complement receptor; MBP, major basic protein; ECP, eosinophilic cationic protein; ECP, eosinophilic peroxidase; NGF, nerve growth factor.

In addition to neutrophils, granulocytes include eosinophils, basophils and mast cells (**Figure 2**), which are rare in the study of *C. trachomatis* now. Eosinophils have unique chemokine receptors on their surface, and Vicetti Miguel et al. revealed in mouse experiments that IL-4-producing eosinophils promoted endometrial stromal cell (ESC) proliferation during primary *Chlamydia* infection, thereby repairing endometrial tissue induced by genital pathogens (78). In addition, a recent pathological study on endometriosis and endometritis revealed a significant increase in not only eosinophils but also basophils (79). And statistically, histological analysis of sexually transmitted infections caused by *C. trachomatis* in gay men presenting with proctitis revealed a scarcity of eosinophils (80). Mast cells are less frequent in studies of *C. trachomatis* infections and more frequent in allergic diseases. In a recent study of *Chlamydia pneumoniae (C. pneumoniae)*, Norika Chiba et al. constructed mast cell-deficient mice (Wsh) and then found that the lungs of Wsh mice were more efficient at clearing *Chlamydia* pathology than those of WT mice. This suggests that the presence of mast cells exacerbates the inflammatory response and increases mortality, because it promotes the infiltration of immune cells into the air and provides a more favorable environment for *C. pneumoniae* to multiply (81).

## 4 Evasion of innate lymphoid cells [ILCs]

### 4.1 NKs and ILCs

The predominant innate lymphoid-like cells are natural killer cells (NKs), which do not have specific antigen recognition receptors like TCR and BCR or pattern recognition receptors like Mφ and DC. In the case of infection, killer activated receptors (KAR) and killer inhibitory receptors (KIR) on the surface of NK cells bind to target cell surface ligands (MHC-I), resulting in the deletion or downregulation of MHC-I molecules and abnormal expression or up-regulation of non-MHC-I molecules. At this point, the inhibitory signal is absent and the activating signal predominates, thereby initiating killing (82). MHC-I is down-regulated, and MICA (MHC class I-related protein A) is up-regulated on cells infected by *C. trachomatis*, which can be recognized by NK cells. Moreover, specific combinations of NKG2D (NK cell-activated receptor) and MICA alleles may promote escape of *C. trachomatis* from NK cell-mediated immune responses more effectively than others in different individuals, which may be a factor contributing to individual prognostic differences in female genital tract infections (83).

In addition to NK, other innate lymphoid cells that do not express specific antigen receptors and whose activation does not depend on recognition of antibodies are named ILCs, which are classified as ILC-1, ILC-2, and ILC-3. ILCs have no direct killing capacity, and their response depends on the activation of cytokines released by other cells, as they have only cytokine receptors. Activated ILCs also release corresponding cytokines to amplify the intensity of the previous immune response to perform an immunological function (84). Both ILC-1 and ILC-3 can secrete IFN-γ. IFN-γ produced by a variety of cells is known to be effective in clearing different sites or species of *Chlamydia*. IFN-γ+ILC-3 plays an important role in regulating colonization of the colon by *Chlamydia muridarum* and inhibiting endometrial chlamydial infection. IFN-γ produced by circulating cells such as NK cells and NKT cells prevents the spread of *Chlamydia* (85). Hong Xu and colleagues demonstrated that mouse ILCs significantly promote endometrial innate immunity in adaptive immunodeficient mice through interferon-dependent mechanisms, and that the role of ILC-3 is more important (86) in mice with *C. trachomatis* inoculation.

In addition to the frequently studied models of reproductive tract infection, gastrointestinal *C. trachomatis* is currently considered a seed bank for recurrent reproductive tract infections. Koprivsek et al. suggested in a murine model of *Chlamydia muridarum*-induced gastrointestinal infection that *Chlamydia* could evade IFN-γ from ILC-3 but not NK and maintain its long-term colonization in the colon (87). Furthermore, IFN-γ induced downregulation of c-Myc, a key regulator of host cell metabolism, in a STAT1-dependent manner, suggesting that c-Myc expression rescued the survival of *C. trachomatis*. IFN-γ caused the persistence of epithelial *Chlamydia* through infiltrative secretion by T cells and NK cells, as found in a pathological study of trachoma scarring (88, 89). Concerning therapy, it has been documented that NK cells are potential therapeutic targets in inflammatory bowel disease (IBD), atherosclerosis (AS), pulmonary arterial hypertension (PAH), and other inflammatory diseases (31). “NK therapies” are known to be widely used in cancer treatment. A nanoparticles (NP) therapy against the *C. trachomatis* antigen: Nanoparticles composed of poly (D,L-lactide-co-glycolide) (PLGA) have attracted much attention because of their biodegradability, biocompatibility and good colloidal stability. Co-delivery of *C. trachomatis* MOMP and immunostimulant (IS) with PLGA particles can prevent systemic adverse effects of immune boosters and activate dendritic cells and natural killer cells, thus enhancing the therapeutic effect of antigen-loaded PLGA particles (90).

## 5 Evasion of innate-like lymphocytes [ILLs]

### 5.1 NKT and γδT

Both Natural killer T cells (NKT cells) and γδ T cells belong to the atypical T cell family, and antigen recognition by these atypical T cells is not restricted by MHC class I and class II molecules (91). NKT cells are lymphocytes that express both the NK cell surface marker CD56 (NK1.1 in mice) and the T cell surface marker TCRαβ-CD3 complex. NKT cells are usually divided into type I and type II. Type I NKT cells are called semi-invariant NKT cells (iNKT) and recognize glycolipid and lipid antigens presented to them by the CD1d molecule, which respond rapidly to danger signals and pro-inflammatory cytokines (92). However, *C. trachomatis* infection can downregulate the CD1d molecule in human penile urothelial cells, which is associated with the CPAF protein. Activated type I NKT can promote the maturation and differentiation of DCs into CD8+/CD103+ DCs, which can further activate T cell differentiation into functional Th1 or Tc1-like peptide antigen-specific T cells. Activated antigen-specific CD4^+^Th1 and CD8^+^Tc1 can suppress bacterial infection (93, 94). However, CD1d-restricted NKT cells can regulate the immune response to chlamydial infection and cause immunopathological damage. Recent studies have shown that wild-type (WT) female mice have a significantly higher chlamydial burden than CD1d-/-(NKT-deficient) mice, suggesting that NKT cells delay chlamydial clearance and exacerbate immunopathology such as tubal effusion and obstruction. In contrast, there is no significant difference in the severity or incidence of tubal effusion in Jα18-/-(iNKT-deficient) mice compared with WT controls. Thus, non-invariant NKT cells have an immunopathogenic role in urogenital tract chlamydial infection (95). This partly explains a seemingly contradictory earlier study that NK T cells triggered pathological Th2 responses during chlamydial infection (96). It has also been shown that the activation of NKT triggers a pathology also associated with disruption of the CXCL13-CXCR5 axis. In this process, activated NKT cells increase chronic inflammation in the upper genital tract of mice by secreting cytokines or chemokines to recruit neutrophils and dendritic cells (97). These results suggest that NK T cells show protective Th1 immunity and pathological Th2 immunity in their role against *Chlamydia*.

γδT cells are known to bridge the gap between innate and adaptive immunity. γδT cells represent a small proportion of the population and are mainly distributed in mucosal tissues such as the peritoneal cavity, intestine and lung. They are the first to be recruited for mucosal infections. Experiments have shown that IL-17A plays a protective role against *Chlamydia pulmonary* infection, and it is produced rapidly but transiently by γδT cells in the early stages and mainly by Th17 in the later stages. Although the depletion of γδT cells led to a decrease in CD80 expression and an increase in IL-10 production by DCs, it had little effect on IL-12 production. There was no effect on type 1 T-cell responses after γδT cells depletion. In contrast, the decrease in IL-1α was more pronounced, suggesting that γδT cells could play a supportive but non-essential role in host defense against *Chlamydia pulmonary* infection (98, 99).

### 5.2 B-1

B cells are divided into B-1 cells, which mediate the innate immune response, and B-2 cells, which mediate the adaptive immune response. Depending on the presence or absence of surface CD5 molecules, B-1 cells can be further subdivided into B-1a (CD5+) and B-1b (CD5-). B-1 is mainly found in the peritoneal cavity, pleural cavity and lamina propria of the intestine. Most of the B cells are restricted to B-1 cells, which can be activated by TI-Ag (bacterial LPS, *etc.*) and produce antibodies with low specificity and do not produce memory cells. Mouse B-1 cells are thought to be the primary producers of natural antibodies to IgM (100, 101), which are independent of foreign antigen stimulation and mediate mucosal immunity below the mucosal lamina propria. Furthermore, in response to antigenic stimulation, it is estimated that 50% of serum IgA and IgG3 are also derived from B-1a cells (101). Lack of BCR diversity on the surface of B-1 cells and reconstitution of IgM-BCR complexes may explain the antigen-specific responses of self-reactive B-1 cells in response to infection by various pathogens, including bacteria, viruses, fungi and parasites (102). At the onset of infection, B-1a cells also spontaneously secrete IL-10 stimulated by lipopolysaccharide (LPS), GM-CSF and IL-3 (103). These natural antibodies and cytokines can protect the host from infection or reduce bacterial burden. In addition, B-1a cells are efficient antigen-presenting cells that provide effective signals to T cells through the co-stimulatory molecule CD80/CD86, which is constitutively expressed on B-1a cells (104). B-1 cells play an important role in assisting M1-type macrophages in the killing of Encephalitozoon cuniculi and in reducing its immune escape mechanism (105). In conclusion, the study of trends in B-1 cells and inflammation may lead to a paradigm shift toward sustainable treatment of various inflammatory diseases (101). *C. trachomatis* infection is an inflammatory disease and it is known that *Chlamydia* can colonize the gastrointestinal tract for long periods of time (106). Although B-1 cells have been little studied in *C. trachomatis*, this may, provide new ideas for *C. trachomatis* research.

## 6 Conclusions and perspectives

**Figure 3:**
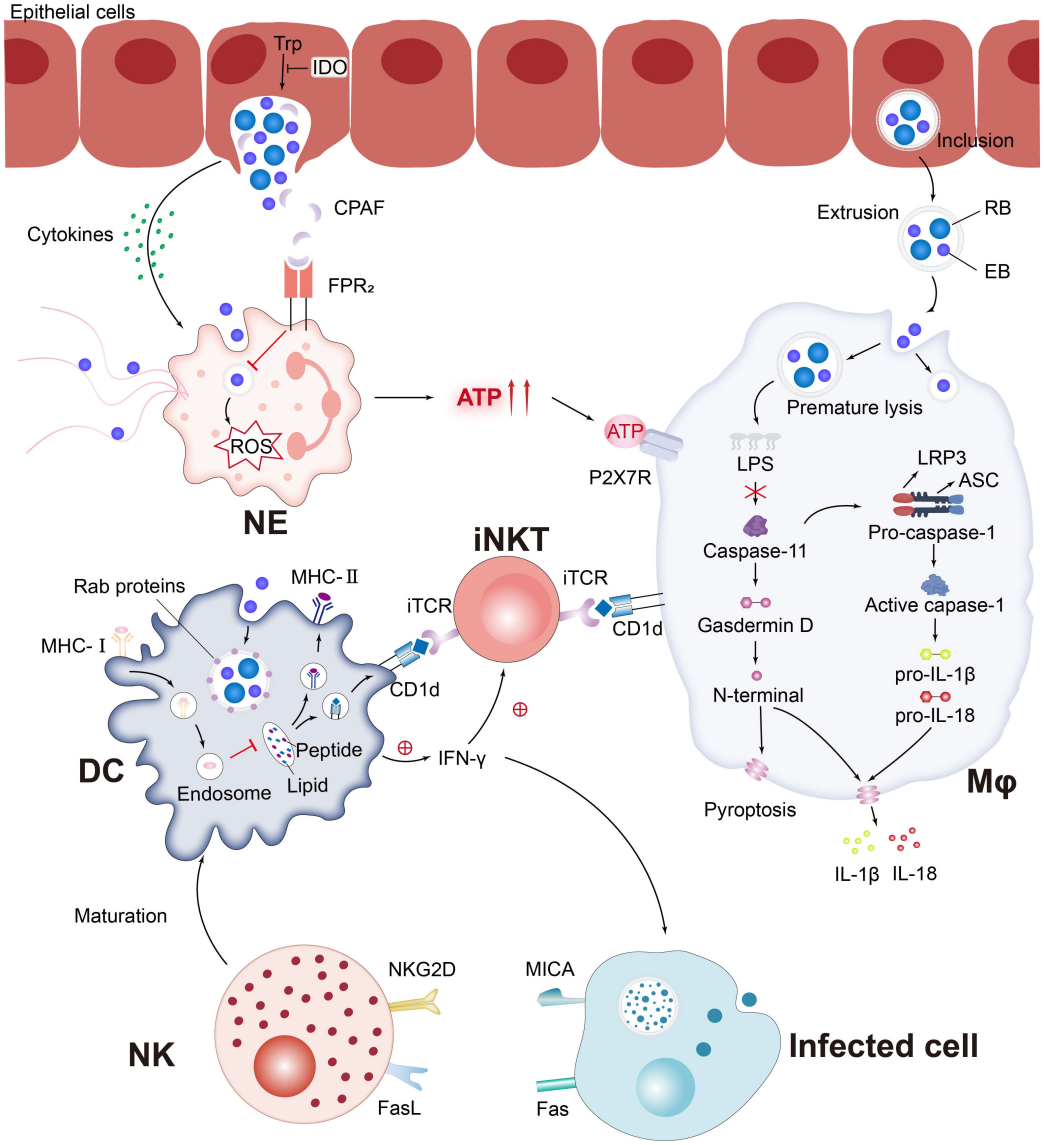
*C. trachomatis* evades the pursuit of innate immune cells. Pro-inflammatory cytokines secreted by *C. trachomatis*-infected cervical epithelial cells attract innate immune cells to the site of infection. *Chlamydia* protease-like activating factor (CPAF), which targets the cleaved NE surface receptor formyl peptide receptor 2 (FPR2), blocks the formation of neutrophil extracellular traps (NETs) and inhibits downstream reactive oxygen species (ROS) production, which paralyzes murine polymorphonuclear neutrophil (PMNs) activation. *Chlamydia*-infected NE produces elevated levels of extracellular ATP, adenosine triphosphate (ATP) that binds to P2X purinocreceptor 7 (P2X7R) and activates the NLRP3 inflammasome, thereby contributing to macrophage-associated immunopathology. *Chlamydia* is released from epithelial cells by extrusion and then forms extrusions that are taken up by Mφ. Interferon-induced GTPases are known to promote inclusions ubiquitination, leading to premature inclusion lysis. Bacterial lipid antigens are presented to iNKT cells via CD1d molecules on the surface of Mφ and DC. Activated NKT and NK promote DC maturation through the release of IFN-γ and positive feedback from cell-to-cell interactions. Rab proteins involved in the DC endocytic cycle are recruited around the inclusions and impede MHC-I intracellular trafficking. Notably, MICA upregulation occurs in parallel with MHC class I downregulation, affecting the sensitivity of *C. trachomatis*-infected cells to NK cell activity.

Currently, the interaction between various innate immune cells and *C. trachomatis* is still a challenging research topic, and both compete and promote each other in the course of a long-term battle (**Figure 3**). The complex set of mechanisms involved in the killing of innate immune cells against *Chlamydia* infection and *Chlamydia* immune escape from host cells can be broadly classified as follows:

Firstly, resisting the phagocytic bactericidal effect of the phagocyte. *C. trachomatis* resists phagocytes through secreting specific effector proteins that evade the capture of NETs and prevent the activation of Mφ. In addition, the ability of nascent inclusions to evade fusion with phagocytic lysosomes is also related to these effector proteins, for example, IncE disrupts retromer and lysosome function by binding SNXs 5 and 6 (sorting nexin). However, the mechanism is not clear (38).

Secondly, blocking the activation of the lymphocytes. The persistent presence of *C. trachomatis* in innate immune cells may due to the pressure exerted by T lymphocyte-mediated immunity, which is the primary defense mechanism of the host against *C. trachomatis* infection. This prompts *C. trachomatis* to interfere with host antigen presentation by downregulating MHC molecules on the surface of target cells, including downregulation of MHC class I molecules that impede CD8^+^ CTL activation and downregulation of IFN-γ-induced MHC class II molecules that impede CD4^+^ T lymphocyte activation.

Thirdly, reproducing in immune cells (anti-apoptosis). *In vitro* experiments have shown that the surface structures of EB, like LPS and certain proteins, promote endocytosis of *Chlamydia* by susceptible cells (25). For example, the pore protein OmpA of the *C. trachomatis* outer membrane and the plasmid-encoded Pgp3 respectively inhibit apoptosis by targeting the pro-apoptotic proteins Bax and Bak (107), or block the activation of the apoptotic signaling pathway (108), which facilitates the pathogen to use the host cells for nutrition to multiply in and survival. In addition, *C. trachomatis* converts from RBs to AB (aberrant body) by changing the expression of HSP60, outer membrane proteins and LPS when it enters a crypt-infected state under the influence of external stresses (antibiotic use, iron deficiency or co-infection). This process is convenient for *C. trachomatis* to escape the anti-infective immune response of the host. ABs can be converted back to RBs in an active state, then RBs transformed into infectious EBs and released from the target cells when the external pressure is reduced or removed. This release mechanism is associated with the CPAF protein (32, 109).

Fourth, inducing the immune cells to apoptosis or directly killing immune cells. Host exposure to *Chlamydia* infection is known to exhibit high levels of metabolism, including sugar metabolism, nucleotide metabolism, *etc.*, which is attributed to the reproduction-dependent nature of the bacteria. Moreover, *Chlamydia* infection causes excessive production of reactive oxygen species (ROS), causing oxidative DNA damage, resulting in single-strand breaks and even double-strand breaks, which can severely damage host cells. For example, *Chlamydia* not only causes macrophage foam (110), but also stimulates macrophages to produce TNF-α and induce apoptosis in neighboring T cells (111, 112). Hydrogen sulfide (H2S)-mammalian endogenous signaling gas transmitter is reported to exert protective effects on various innate immune cells against damage from ROS, immune or inflammatory hyperactivation, and also to control differentiation, maturation or polarization of immune cells (e.g. M2 polarization of macrophages) (113).

Nowadays, there are more studies on the interaction between *C. trachomatis* and classical innate immune cells, such as Mφ, NE and DC, but few studies on ILCs and ILLs. And the detailed aspects of how *C. trachomatis* evades innate immune cell pursuit need to be explored in depth by further techniques. Cellular immunotherapies have been reported to be emerging in the field of cancer (114), but this has rarely been studied in pathogenic infections. Therefore, an in-depth understanding of the interaction between *Chlamydia* and innate immune cells will provide further therapeutic interventions to combat this intractable epidemic.

## Author contributions

XW: Writing – review & editing, Writing – original draft. HW: Writing – review & editing. CF: Writing – review & editing. ZL: Writing – review & editing.
